# Towards near real-time, monthly fossil CO_2_ emissions estimates for the European Union with current-year projections

**DOI:** 10.1016/j.apr.2021.101229

**Published:** 2021-12

**Authors:** Robbie M. Andrew

**Affiliations:** CICERO Center for International Climate Research, Postboks 1129 Blindern, 0318, Oslo, Norway

**Keywords:** European Union, CO_2_ emissions, Short-term projection, Monthly emissions

## Abstract

With the international goals of the Paris Agreement and the growing number of time-bound national goals for emissions reductions, reliable estimates of CO_2_ emissions are becoming more and more important. In particular, reducing the time lag of these estimates and producing short-term projections are gaining importance as the remaining time until mitigation deadlines becomes shorter. The Global Carbon Project has been producing a current-year projection of global CO_2_ emissions since 2012, introducing a sub-projection for the European Union in 2018. The success of this EU projection has been variable, and in this article I explore how the projections in 2019 were made along with some of the reasons why the projections have high uncertainty and bias. About 84% of the total error in the projection of EU emissions in 2019 was because of a poor projection for coal consumption, which was a result of poor estimates of sub-annual observations, a misunderstanding of conflicting information, and poor assumptions applied to the remainder of the year. The correction of the errors identified here will go some way to improving future short-term projections of the European Union's CO_2_ emissions, paving the way for a low-maintenance, operational system.

## Highlights

• Few European countries report estimated emissions with a low temporal lag.
• A method used for projecting current-year CO2 emissions in the EU is closely analysed.
• The projection for 2019 was relatively poor, largely because of coal.
• Corrections are suggested that would have greatly improved the projection.

## 1 Introduction

With the increasing focus and effort on reducing emissions of greenhouse gases, there is a growing need for more reliable data as well as data with a lower publication latency. The generation of short-term projections takes this one step further, permitting even earlier feedback of results to the policy environment.

Initial estimates of annual global carbon dioxide (CO_2_) emissions are generally delayed by some months as data must be gathered from numerous sources and then verified. For the European Union (EU), ‘early estimates’ are published by Eurostat – the statistical office of the European Union – in May of the following year (Eurostat, 2020c). EU member states must report by 31 July approximate emissions inventories for the previous year (Y-1), but any methodological revisions are not applied to earlier years, and these are generally not made public before October. By 15 January member states must report preliminary emissions inventories for all years through Y-2, and by 15 March final emissions inventories for all years through Y-2 (EU, 2018, 2013). These last reports are made public on 15 April when they are published by the United Nations Framework Convention on Climate Change (UNFCCC).

The Global Carbon Project (GCP) has been producing projections of the current year's global fossil CO_2_ emissions since 2012 (Le Quéré et al., 2013). At first the method used was relatively straightforward, using a simplified Kaya decomposition (Raupach et al., 2007) applied at the global level, with the expected emissions intensity of the economy multiplied by the expected size of the economy. The expected emissions intensity was taken as an extrapolation of the previous 10 years' emissions intensities, effectively assuming this intensity would continue to improve at the same rate. The size of the economy was taken directly from forecasts of global growth of gross domestic product (GDP) from the International Monetary Fund (e.g., IMF, 2019).

This method worked well for several years as the world's economy was moving in a relatively consistent direction, but in 2014 China's emissions pathway changed while its economy continued to grow, and the projection method failed (Le Quéré et al., 2015a, 2015b). As a direct result, the method was improved to project China and the US – the two top emitters – separately, with the rest of the world projected using the intensity approach. The US projection was taken directly from an existing projection made by the US Energy Information Administration (EIA, 2015), with additional information on cement production, while the projection for China was derived from the limited available sub-annual, national energy production, trade and stocks data and cement production data (Le Quéré et al., 2015a).

In 2017 the GCP began making a separate projection also for India, and in 2018 for the European Union (Le Quéré et al., 2018a, 2018b). The work on low-lag monthly estimates for India became very detailed and was published separately by Andrew (2020), while the work on the EU has not yet been published in detail. The projection method for the EU relied partly on extrapolation of seasonal trends using the monthly data by fuel category.

In 2020, with the global COVID-19 pandemic, several independent estimates of global and EU sub-annual CO_2_ emissions were produced, and these were included in the GCP's projection of 2020 emissions (Friedlingstein et al., 2020). With the breakdown in the normal seasonal pattern, the method previously used to project forward the last few months of the year was no longer viable, and an alternative, simpler method was used.

The purpose of this article is to investigate and explain the performance of the projection of fossil CO_2_ emissions in the EU for the year 2019, and thereby to pave the way for improvements in future projections. Given the strong dependence of the projection on low-lag estimates of sub-annual emissions, this paper will next look at existing official and unofficial estimates of sub-annual national CO_2_ emissions, then present the method used to generate monthly observations in 2019, and in turn the method used to project those observations forward to obtain an estimate for the full year. This is followed by a retrospective analysis of how the observation and projection methods performed, and a concluding section.

### 1.1 Existing official estimates of historical sub-annual emissions

Eurostat has been publishing early estimates of annual CO_2_ emissions in the EU since at least 2012 (Eurostat, 2013, 2014, 2020b; Herold et al., 2017, 2018; Scheffler et al., 2020). These provide first estimates of emissions within five months of the end of the reference year, and make use of monthly energy data, without resulting in monthly emissions estimates.

While there is currently a lack of EU-wide official estimates of sub-annual emissions, some member countries do produce such estimates. National estimates can be produced either as part of environmental accounts, in which case coverage aligns with national accounts (e.g., GDP) and therefore include emissions from all economic activity, or they can be territorial emissions, in which case only emissions occurring within the country's territory are included. Territorial emissions are consistent with the UNFCCC's reporting requirements (Rypdal et al., 2006), while environmental accounts are not.

In late 2015, Statistics Sweden began publishing quarterly environmental accounts of greenhouse gas emissions, with estimates beginning in 2008 and a current lag of about four months (SCB, 2016, 2020). Sweden's Environmental Protection Agency did publish quarterly emissions on a territorial basis, but these are apparently on hold as methodological revisions have created a discontinuity in the underlying transport fuel data (pers. comm., SCB, January 12, 2021; Naturvårdsverket, 2020).

Since about 2010, Statistics Netherlands has published quarterly changes in CO_2_ emissions as part of its environmental accounts with a lag of three months (again, with an economic boundary rather than a territorial boundary) (van Rossum and Schenau, 2010; CBS, 2020b). While quarterly changes are published, absolute levels are not. In 2020, Statistics Netherlands, on request from the Ministry of Economic Affairs and Climate, started to investigate the production of monthly and quarterly estimates of greenhouse emissions on a territorial basis (CBS, 2020c), with the first release in early 2021 (CBS, 2021). This development is likely an outcome of the “Urgenda climate case” in 2015 (upheld by the Supreme Court in 2019 (Hoge Raad, 2019)), which resulted in a legal obligation for the Netherlands to reduce its territorial emissions in 2020 to 25% lower than the 1990 level (CBS, 2020a). This very short timeline necessarily requires high-frequency, low-lag estimates on which to base mitigation decisions.

The United Kingdom's Department of Energy & Climate Change began publishing quarterly territorial greenhouse gas emissions estimates in 2013 (DECC, 2014, 2015), and these were subsequently continued by the Department for Business, Energy and Industrial Strategy (BEIS), but frequent publication ceased in 2016 following user consultation, and quarterly time-series are now released annually at the same time as the provisional annual time-series (BEIS, 2016). The reason for their estimation is to facilitate the earlier estimation of provisional annual estimates.

Elsewhere in the world, Australia has been publishing quarterly territorial greenhouse gas inventories since August 2009 (DoCC, 2009), and the United States first published monthly territorial CO_2_ emissions in the very same month (EIA, 2009). New Zealand began publishing quarterly estimates of emissions in 2021, although these are part of its environmental accounts rather than territorial estimates (Statistics NZ, 2021).

The case of Sweden's interrupted publication of territorial emissions estimates indicates an important lesson: while sub-annual activity data (energy consumption, etc.) may be officially published, this does not guarantee their quality, with annual data generally seen as most important both because these are reported internationally and because they are used in national decision-making. This importance leads to significantly more effort in producing robust statistics at the annual level, evidenced by the far more complex methodologies used for annual statistics than for the few existing sub-annual statistics discussed above. Indeed, the methods used by these countries to produce their sub-annual estimates generally include quite simple assumptions, particularly the scaling of the most recent year's annual estimate by sub-annual activity data.

The Swedish development was driven by the statistics agency, rather than being specifically requested by other government ministries and departments (pers. comm., Statistics Sweden, January 21, 2021). While the Swedish publication of quarterly emissions estimates is partly justified by “[making] it possible to monitor current emissions trends” (SCB, 2016, p. 10), it is not yet clear whether any decisions are made as a result of this monitoring. The recent case of the Netherlands is currently exceptional, although may be a sign of things to come, with potentially more countries finding the need for sub-annual emissions estimates to better track short-term targets. However, rather than quarterly estimates being useful in themselves, one of the stated and more likely valid rationales for producing quarterly estimates is so that preliminary annual estimate can be produced significantly earlier than standard final estimates (SCB, 2016).

### 1.2 Existing third-party estimates of historical sub-annual emissions

The estimation of sub-annual emissions by third parties goes back a few years further, possibly starting with Blasing et al. (2005), who estimated monthly CO_2_ emissions in the USA from consumption of fossil fuels, using the readily available statistics published by the EIA. Blasing and Kimberly (2007) later added estimates for cement production and flaring of natural gas.

Shortly afterwards, Gregg et al. (2008) published approximate estimates of monthly CO_2_ emissions in China using monthly production data. For example, China's annual emissions from coal as estimated by the Carbon Dioxide Information Analysis Center (CDIAC) were spread over months using weighted data on production of thermal electricity, steel, and coke along with the value of industrial outputs.

The foregoing work was then extended by Andres et al. (2011), who produced the first estimates of monthly global CO_2_ emissions using proxy data for 21 high-emitting countries and then mapping countries without data to similar countries that did have data.

As mentioned earlier, Andrew (2020) presented monthly estimates of India's fossil CO_2_ emissions, and calendar-year estimates from these have since been incorporated into the Global Carbon Project's fossil CO_2_ database (Friedlingstein et al., 2020).

The year 2020, with the global pandemic, saw an increase in interest in low-latency emissions estimates. Liu et al. (2020) developed Carbon Monitor, a daily, global CO_2_ emissions dataset with country-level estimates for 12 countries. Emissions are estimated largely using proxy data, which are available with lower lag than comprehensive energy statistics. For example, emissions from road transport across the world were calculated using a commercial traffic-congestion index for over 400 cities combined with a regression relationship derived from data in Paris (Liu et al., 2020). Le Quéré et al. (2020) also developed a method for estimating daily, global CO_2_ emissions, based on much of the same underlying activity data, but using it to parameterise a pandemic-related ‘confinement index’, which could then be applied to all countries.

The atmospheric inversion community has also been developing sub-annual emissions, which they call temporal profiles, although these are generally averaged over several years rather than being specific to each year (e.g., Guevara et al., 2021). This modelling community uses gridded emissions, often down-scaled from annual or monthly national emissions estimates. For example, the data and method used by Andres et al. (2011) was used by Andres et al. (2015) to generate a monthly, gridded fossil CO_2_ emissions dataset. Further examples include: Olivier et al. (2005), who presented monthly gridded emissions based on EDGAR data; Crippa et al. (2020), who presented monthly temporal profiles for use in the temporal disaggregation of annual emissions grids; Oda et al. (2018), who averaged the intra-annual emissions from CDIAC to obtain a temporal emissions profile that they apply to all years; and Janssens-Maenhout et al. (2019b), who published EDGAR v4.3.2 monthly gridded data for the year 2010 (Janssens-Maenhout et al., 2019a).

## 2 Methods

### 2.1 Historical estimates

The general approach used is to collate observational data on the consumption of solid, liquid, and gaseous fossil fuels for each country in the EU and convert these to CO_2_ emissions using standard emission factors. This energy-based approach is chosen because comprehensive energy data are collected primarily on the basis of production, trade and transformation of energy products, which tend to be reported via robust protocols. In contrast, energy on how much sectors, companies, and individuals consume is often derived from a combination of direct reporting and surveys. Either approach – by fuel type or by sector – could be used, but we chose the former, also because that matches the format of the emissions data produced by the GCP, which is historically based on data from the Carbon Dioxide Information and Analysis Center (CDIAC), which is by fuel type.

Monthly production and consumption data for all three fuels are provided by Eurostat with a relatively low lag (Eurostat, 2021a). Eurostat and IEA submission deadlines are both approximately the 25th of each month for the month before last, M-2: “Administrations are to provide monthly oil and gas data for the month before last (M-2) on the 25th of each month to the IEA and within 55 calendar days after the end of each reference month (M-2) to Eurostat, i.e. March data is to be submitted on the 25th of May.” (OECD et al., 2020, p. 1). Eurostat's monthly collated dataset is updated significantly more frequently than once each month.

In practice, there are always some countries that delay their reporting, so full EU coverage is rarely available for M-2. For oil and natural gas, administrations also report monthly data to the Joint Organisations Data Initiative (JODI) for both M-2 and M-1, giving in many cases one month more recent data than is reported to Eurostat (JODI, 2021b, a). However, this is possible only at the expense of much less detail: while the Eurostat monthly oil survey lists 19 petroleum products, JODI has only eight; JODI does not have biofuels explicitly reported; JODI has only five energy flows, while Eurostat has 16 or more, depending on how they're counted (JODI, 2020). Moreover, JODI releases these data publicly typically about four weeks after the submission deadline, such that the most recent data are always M-2. Comparison with Eurostat data suggests that historical JODI data are not often – if ever – revised.

### 2.2 Method used in 2019

Despite the 2016 UK vote to leave the EU, and the official announcement in 2017 (Walker, 2021), the UK was still a member of the EU in 2019 and still bound by its regulations, and the UK was therefore included in our analysis of EU emissions.

The method aims to produce total fossil CO_2_ emissions, built up in the same way as the Global Carbon Budget, from estimates of the three major fossil-fuel groups – coal, oil, and gas – and emissions from carbonate decomposition in cement production. The same approach was used in 2019 for both oil and natural gas, with Eurostat energy data used in preference because of its extra detail, separation of biofuels, and that historical data are subject to revision, in contrast to JODI data. The analysis was performed with data downloaded from Eurostat on November 4, 2019, and the JODI October 2019 editions. The flow “Gross inland deliveries observed” (GIDO) was used on the assumption that it is generally more reliable to use countries’ best estimates of national consumption than “Gross inland deliveries calculated” (GIDC), which is calculated from other terms. Energy data are obtained in energy units (TJ) in gross calorific value (GCV), which is converted to net calorific value (NCV) by using the factor suggested by the Intergovernmental Panel on Climate Change (IPCC), 0.90 (Eggleston et al., 2006). The final month of data varies by country and flow.

The JODI dataset was then used to extend the Eurostat data through August 2019, with the number of months of extension depending on the final month of data for each country in the Eurostat data. Growth rates of observed consumption in JODI were applied directly to the Eurostat consumption data in NCV terms. Finally, the energy consumption data were multiplied by default IPCC emission factors by fuel type (Gómez et al., 2006; see supplementary material) to obtain estimates of CO_2_ emissions by month and country, and these were summed to give EU emissions by fuel type. Lastly, the emissions from each oil fuel type (excluding non-energy products such as bitumen) were added to give total EU emissions from combustion of oil and oil products.

For coal, both the calculated and observed gross inland deliveries from Eurostat monthly energy data were used, giving slightly different results. Eurostat's monthly data had longer data lags for some important countries, with the database as downloaded on the November 4, 2019 providing data for Poland and Germany – the EU's two biggest consumers of coal – only through May and July, respectively. Projecting the remainder of the year based on such a short observation period would have introduced significant uncertainty, so an alternative approach was used in parallel.

Electricity generation data is reported by transmission operators to the European Network of Transmission System Operators for Electricity (ENTSO-E, 2021). These data are reported in sub-daily intervals with very short latency, but since oil and gas data were only available monthly, electricity data were also aggregated to monthly statistics. Analysis indicated some periods of missing data in the coal series for the UK, so these were supplemented using data from Elexon (2021). This dataset covered 89% of the total gross generation from solid fossil fuels (including peat and oil shale) in 2018 as reported by Eurostat (2021b). At the time of analysis, data were available through October 2019.

To represent other uses of coal in the EU, monthly steel production data were used as a proxy for all industrial uses (World Steel, 2020). These data have a lag of less than six weeks, and at the time of analysis, data were available through September 2019. The change in the amount of electricity generated from solid fossil fuels was combined with the change in non-electricity consumption weighted by the share electricity generation and combined heat and power (CHP) in total EU coal consumption in energy terms, 70% in 2016, the most recent available at the time the work was performed (IEA, 2018).

I now summarise this method concisely using equations. For coal and oil, Eurostat's monthly data are reported in mass units (kilotonnes), so must first be converted to energy units before calculating emissions, as in the following equation:

(1) $$CO_{2}=\underset{f,c}{\sum }F_{f}C_{f}M_{f,c}$$

where $M_{f,c}$ is the consumption of fuel f in country c in mass units from flow “Gross inland deliveries”, $C_{f}$ is the default IPCC (NCV) energy content of fuel f, and $F_{f}$ is the default IPCC emission factor of fuel f.

Natural gas is reported in energy units, but in gross calorific value, so must be converted to net calorific value before applying the IPCC default emissions factor, which is in NCV terms, as in the following equation:

(2) $$CO_{2}=\underset{c}{\sum }0.90\times FE_{c}$$

where $E_{c}$ is the consumption of natural gas country c in energy units (GCV) from flow “Gross inland deliveries”, and F is the IPCC default emission factor for natural gas.

The extrapolation of oil and natural gas energy consumption data using JODI can be represented as:

(3) $$E_{f,c}^{t+1}=\left(1+J_{f,c}^{t+1}\right)E_{f,c}^{t}$$

where $E_{f,c}^{t}$ is the energy consumption of fuel f in country c in month t and $J_{f,c}^{t+1}$ is the growth rate of energy consumption of fuel f in country c in month t+1 according to JODI data.

The extrapolation of coal emissions using electricity and steel data is represented by the following two equations:

(4) $$CO_{2}^{t+1}=G_{coal}^{t+1}\underset{f,c}{\sum }F_{f}^{t}C_{f}^{t}M_{f}^{t}$$

(5) $$G_{coal}^{t+1}=\gamma \left(1+G_{e}^{t+1}\right)+\left(1-\gamma \right)\left(1+G_{s}^{t+1}\right)$$

where $G_{\mathrm{coal}}^{t+1}$ is the growth rate of coal emissions in month t+1, γ is the share of coal emissions from electricity (here 70%), $G_{e}^{t+1}$ is the growth rate of electricity generated from coal in month t+1, and $G_{s}^{t+1}$ is the growth rate of steel production in month t+1.Projections.

With the clear, strong seasonal patterns exhibited by the monthly emissions from oil and natural gas, a method based on trends and seasonality was applied. For emissions from coal, significant changes in trend in recent years mean that an alternative approach was required.

For oil and natural gas, the Holt-Winters exponential smoothing method was used for short-term projections of monthly emissions (Chatfield, 1978; Gardner, 2006; Winters, 1960). Simply put, this iterative method first trains a model with an initial estimate of trend and seasonality, updating this with each new data point by using exponential weighting to combine the effect of the new data point into the existing estimates. For projection, these parameters for trend and seasonality derived from training are then applied to future time periods. The Holt-Winters method has been shown to have equivalent regression, ARIMA, and state-space models (Gardner, 2006).

As discussed above, two approaches were used in parallel for estimation of historical emissions from coal. For the method based on Eurostat energy data, a parsimonious approach was used for projection whereby emissions for the remainder of the year after observations for each country were assumed to be equal to the same period in the previous year. Neither seasonality nor continuation of recent unusual trends was considered suitable, and the given lack of any other information to go by, this ‘low-assumption’ approach was used in combination with high uncertainty. For the method based on electricity and steel production data, the weighted growth rate through the data period was used as the growth rate for the year; this is a quite different assumption to that applied to the Eurostat data. Because two methods were used in parallel, without any clear information at the time on which was superior, the final projection for emissions from coal was taken as a mid-point between the two methods used.

No sub-annual information was found for cement production, which represented 2.4% of EU emissions in 2018 (Friedlingstein et al., 2020), so in the light of recent trends these were assumed to be unchanged (0% growth). Such a rate is more appropriate for developed countries such as in Europe, which have much lower investment in new infrastructure than developing countries such as China.

The final emissions projection for the EU28 in 2019: −10% solid fuels, +0.5% liquid fuels, +3.0% gaseous fuels, +0% cement production. This resulted in a projection for total fossil CO_2_ emissions of −1.7%.

## 3 Analysis of estimates of monthly observed emissions

### 3.1 Natural gas

Natural gas is a relatively straightforward fuel category, with only one fuel and usually reported in both volumetric and energetic units. However, the share used for non-oxidative purposes, such as plastic feedstock, is highly variable. Moreover, some emissions from oxidative uses, such as fertiliser feedstock, are officially reported outside of the IPCC's Energy sector, and some of these products are traded internationally such that emissions do not necessarily occur in the same territory as the natural gas was consumed.

**Fig. 1:**
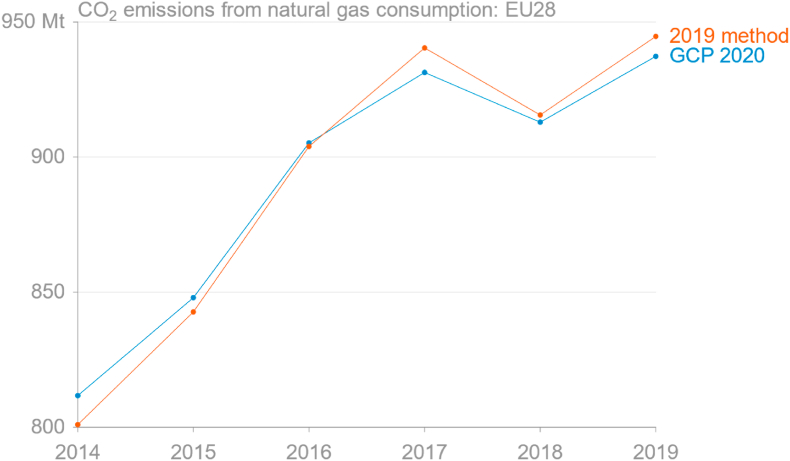
Comparison of estimated annual CO_2_ emissions from consumption of natural gas in the EU from the Global Carbon Project's 2020 release and as aggregated from monthly estimates derived from Eurostat energy data using the 2019 method (source: Friedlingstein et al., 2020; own calculations based on Eurostat, 2021a).

To confirm the consistency of emissions derived from monthly Eurostat energy data with the GCP emissions dataset requires simply aggregating to annual emissions. Fig. 1 demonstrates that applying IPCC-default emission factors to total natural gas demand gives good estimates of total emissions from natural gas, noting that GCP's emissions from natural gas are sourced from official inventory reports and include non-energy oxidation such as emissions from use of artificial nitrogen fertilisers. The root-mean-square error (RMSE) over 2014–19 is 7.0 Mt.

The method used data from JODI to extend the Eurostat data by one month, so it's important to assess how JODI natural gas compare with Eurostat data published later. For natural gas, data reported by JODI match very well those reported by Eurostat for many countries, as is expected given matching flow and product definitions and the same data origins, with differences resulting from the preliminary nature of data submitted to JODI. The data from JODI are therefore suitable for extrapolating one or more months from the Eurostat data.

JODI does not provide a public archive of previous editions of the database but using versions of the database downloaded on different dates allows some analysis of revisions. This analysis (not shown) indicates that while individual countries’ reported natural gas demand can be revised quite heavily from one edition of JODI to the next, at the EU level the maximum revision during 2019 was much smaller, at 0.75%.

### 3.2 Oil

For oil products the situation is much more complicated. First, oil products are divided into a number of sub-products having varying characteristics. Second, a significant proportion of oil products are used for non-energy purposes. Third, consumed diesel is a blend of fossil diesel and biodiesel, and JODI reports only the total. Fourth, some products and flows are reported with significant lags by some countries.

**Fig. 2:**
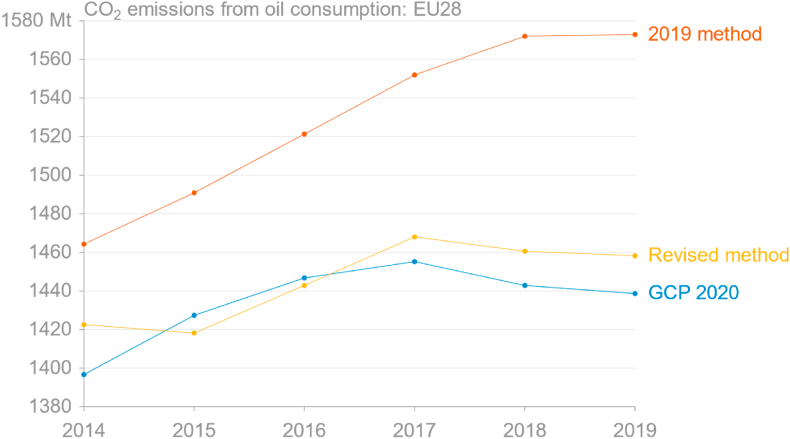
Comparison of estimated annual CO_2_ emissions from consumption of oil in the EU from the Global Carbon Project's 2020 release, as aggregated from monthly estimates derived from Eurostat energy data using the 2019 method, and the 2019 method adjusted for international aviation, naphtha oxidation, and direct use of primary products (source: Friedlingstein et al., 2020; own calculations based on Eurostat, 2021a).

Fig. 2 makes it clear that the method used in 2019 to estimate emissions from oil using Eurostat monthly data not only overestimated absolute emissions, but that the trend is a poor match. That the estimate is too high is not directly a problem, since the goal is to obtain growth rates; that the trend is poor is of greater concern.

Several errors can be identified in the method. One clear error is the use of the flow “Gross Inland Deliveries”, which by definition excludes supply to international maritime bunkers but includes supply to international aviation (OECD et al., 2020), while this is excluded from the GCP's estimates at national – and EU – level. This counterintuitive fact is apparently a direct result of the historically relatively poor availability of data for aviation bunkers (pers. comm., IEA, February 15, 2021). Another error is that the method assumed that all primary products are converted to secondary products, but crude oil and natural gas liquids are primary products that are used directly to some degree. A third is the total exclusion of naphtha, some of which is oxidised, with IEA data suggesting about 20% is used for energy purposes in the petrochemical industry in the EU (IEA, 2019). Correcting these three errors in the method for monthly emissions results in a much closer estimate to the annual estimates reported by the GCP (Fig. 2).

With the revised method, the 2019 annual growth rate in EU emissions from oil derived from the full year's data becomes −0.2%, compared to +0.1% using the 2019 method and −0.3% using data from GCP. It is clear, however, that not all recent years would have performed so well. The RMSE over 2014–19 for the 2019 method was 98.5 Mt, while the RMSE over the same period for the revised method is 16.5 Mt.

Further potential reasons for divergence include the use of IPCC default factors rather than country-specific factors and possible errors in monthly data, particularly earlier in the series. Additional potential avenues of enquiry would likely be uncovered by looking closely at country-level estimates. Eurostat monthly energy data are not perfect, and it has been shown that official reporting is far from complete and correct in all cases, for example with incomplete reporting of international bunker fuels (Herold et al., 2017).

**Fig. 3:**
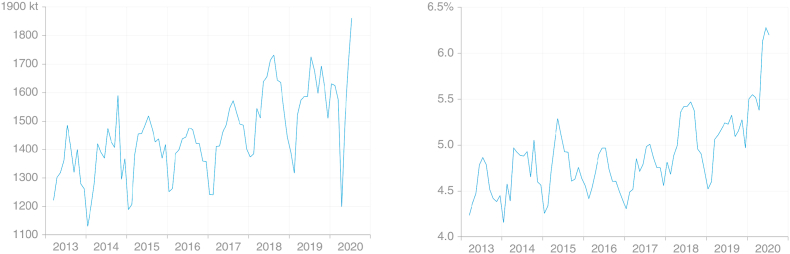
Monthly EU consumption of biofuels (sum of biodiesel and biogasoline), (a) absolute and (b) share of biofuels in total diesel and gasoline (source: own calculations based on Eurostat, 2021a).

The use of JODI data to extrapolate Eurostat data introduces some error given both the preliminary nature of JODI data and the absence of information on how much transport fuel is biofuel. Under current emissions inventory methodologies, biofuels are assumed to be carbon-neutral and therefore not to contribute to emissions in the country where they are combusted. Biodiesel consumption has a seasonal cycle both in relative and in absolute terms: both the amount and share of biodiesel in total diesel change through the year (Fig. 3). Use of JODI data in the 2019 method effectively assumes that the share of biodiesel in total diesel does not change over time.

### 3.3 Coal

Two separate methods were used to produce estimated monthly emissions from coal consumption.

At the time the analysis was performed, Eurostat monthly coal statistics were available through August 2019, but some important countries had not yet reported that month. Of the six largest coal consumers in the EU, only Czechia had reported August at that time, with Germany, France, Romania, and the UK having reported through July, and Poland – the second-largest consumer of coal in the EU – having reported only through May.

**Fig. 4:**
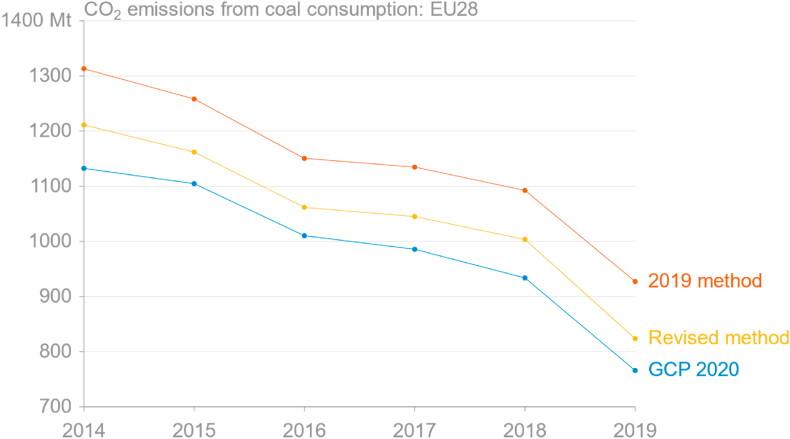
Comparison of estimated annual CO_2_ emissions from consumption of coal in the EU from the Global Carbon Project's 2020 release, as aggregated from monthly estimates derived from Eurostat energy data using the 2019 method, and the 2019 method adjusted for incorrectly double-counted coke-oven coke (source: Friedlingstein et al., 2020; own calculations based on Eurostat, 2021a).

**Fig. 5:**
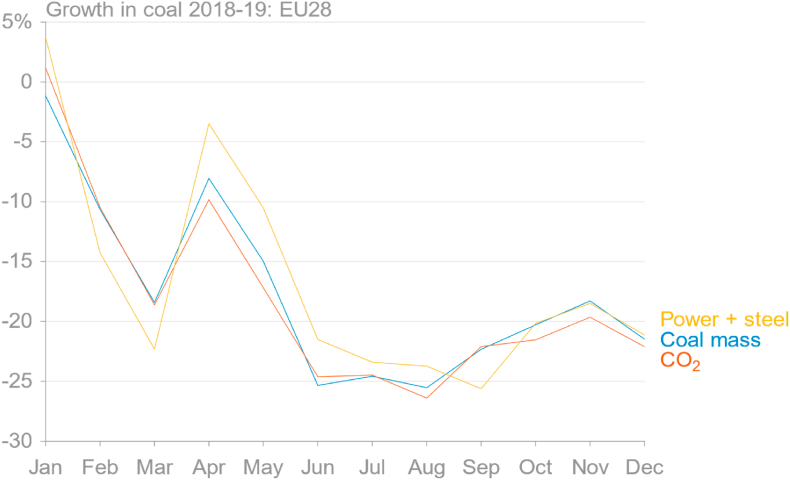
Growth rate of coal and coal emissions by month in 2019 compared to the same months in 2018, with three different approaches. Total of mass of coal is calculated from Eurostat data (GIDC; blue line). CO_2_ emissions here (red line) were estimated from Eurostat data using IPCC default energy contents and emission factors, which account for different grades of coal. The “power + steel” method (yellow line) is described in the text. (source: own calculations based on Eurostat, 2021a; World Steel, 2020; ENTSO-E, 2021).

Taking the calculated emissions directly from the Eurostat-based method and comparing them to GCP's 2020 release demonstrates very good correspondence in trends, although there is a large, relatively constant difference (Fig. 4). The main reason for this is the double-counting of coke-oven coke, the mass for which are already included in reported hard coal deliveries, and when this double counting is corrected (“Revised method”) the series are much closer. The remaining difference most likely results from the method's assumption that all solid fossil fuels are oxidised. Comparison of the growth rates also shows relatively good performance of this method for estimating observed emissions from the consumption of coal, and even better with the revision (Fig. 5). The RMSE over 2014–19 for the 2019 method was 157.8 Mt, while the RMSE over the same period for the revised method is 63.1 Mt.

Estimates were made using both the Eurostat supply-side energy flow “Gross inland deliveries calculated” (GIDC) and the demand-side energy flow “Gross inland deliveries observed” (GIDO), giving differing results. GIDO is only reported for hard coal (Eurostat, 2017; pers. comm., Eurostat, February 2021), making it unsuitable for estimating total emissions from solid fossil fuels, and only the supply-side flow should have been used.

Now that official monthly coal consumption data are available for the full year 2019, the simple approach using electricity generation and steel production data can be assessed. This method does not directly give estimates for monthly emissions, but rather growth rates compared to the same months in the previous year. Fig. 5 shows that the weighted combination of the growth rates of power generation from solid fuels and steel production gives a relatively good approximation of the actual growth rates of both total mass of solid fuels delivered and estimated emissions from the mass data using energy contents and emission factors from the IPCC.

Several core sources of error might be further investigated in future. First, the main data source used for electricity generation ENTSO-E, “represents 42 electricity transmission system operators (TSOs) from 35 countries across Europe,” (ENTSO-E, *no date*) and therefore covers only electricity that is passed through the transmission network, omitting some electricity that is limited to distribution networks. It may, however, be reasonable to assume that most coal generation units are captured by the database. ENTSO-E's coal generation data for the UK were incomplete, and were therefore replaced in the present analysis with equivalent data from Elexon, which were found to be more complete (Elexon, 2021). Coal generation data for the Netherlands are entirely missing in ENTSO-E's 2018 data, but no alternative data source was found, and the country's coal generation is a small share of the EU total.

Second, the simple use of total electricity generation from all solid fossil fuels, rather than weighting these by varying emission factors for different fuels, for example hard and brown coal, introduces some error. Emissions per unit of electricity generated depend on both the calorific value of the fuel used and the efficiency of the power station.

**Fig. 6:**
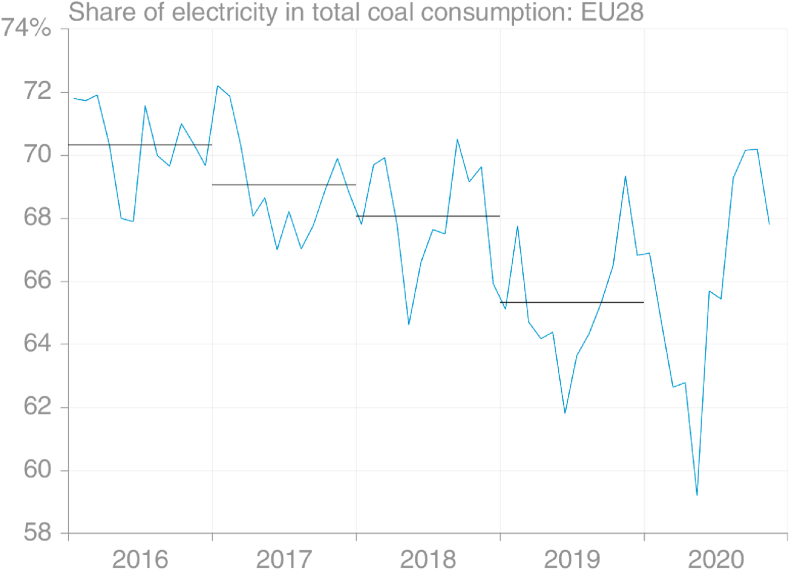
Approximate monthly and annual-average share of electricity in total consumption of coal, EU28 (source: own calculations based on Eurostat, 2021a).

The use of data from two sectors required a weighting based on the share of electricity in total coal consumption along with the assumption that the production of steel was strongly correlated with all non-electricity consumption of coal. Given the declining use of coal for generating electricity in the EU, it would be reasonable to expect that the share of electricity in total coal consumption was dropping, which indeed is borne out by Fig. 6. However, this decline is relatively minor, and the difference between the actual share in 2019, 65%, and share used in the analysis, 70%, would not have had a substantial effect on the projection. But if this were to be improved in future, the seasonal signal in this share – generally lower in summer months – could also be applied, rather than assuming a constant share through the year. However, the large deviations in the electricity-steel method in 2019 during March–May coincide with a period with little change in the share of electricity, suggesting other (or additional) reasons for the deviations.

The two methods for coal emissions – using Eurostat vs. using electricity and steel data – gave divergent estimates, but at the time it was unclear which was more likely to be accurate. It is now clear that both were relatively good as year-to-date estimates, but that the estimate based on Eurostat data simply presented an estimate for fewer months of the year.

### 3.4 Overall

**Fig. 7:**
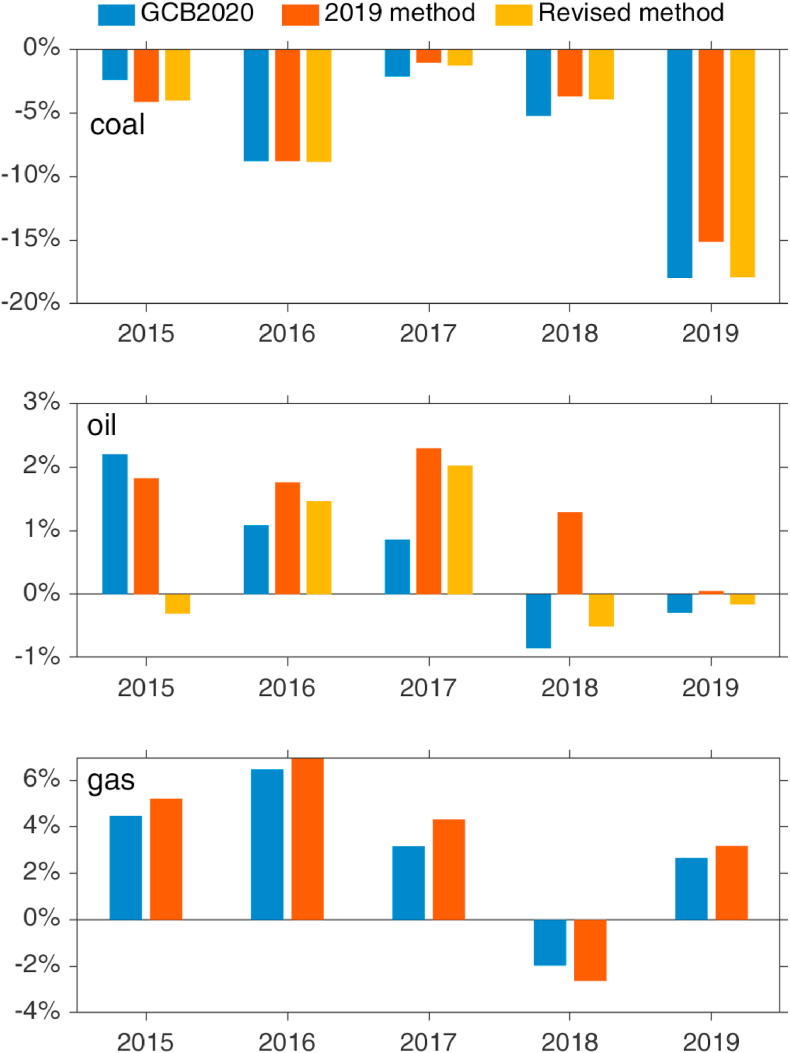
Comparison of annual growth rates derived from the most recent edition of the Global Carbon Budget's fossil CO_2_ dataset with those derived from sub-annual energy and activity data using the ‘2019 method’. For coal and oil, the growth rates from the revised methods are also compared (source: own calculations based on Eurostat, 2021a).

Fig. 7 compares annual growth rates by fuel as estimated using sub-annual data with those published by GCP in 2020.

## 4 Assessment of projections

Emissions in 2019 in the EU were projected to decline by 15.7 Mt, while the GCP 2020 release reported a decline in 2019 of 40.4 Mt (Table 1). The emissions of all components were projected too high (alternatively, the magnitudes of the declines were projected too low), except for cement. Of the total overestimation of 2019 emissions, 84% was due to the error in coal, 13% in oil, and 4% in natural gas.

**Table 1:** Comparison of projected EU28 emissions changes by category with preliminary estimates in GCP's 2020 release.

		Coal	Oil	Gas	Cement	Total
Projection	Relative	−10.0%	+0.5%	+3.0%	0%	−1.7%
	Absolute	−25.2 Mt	+2.0 Mt	+7.6 Mt	0 Mt	−15.7 Mt
GCB2020	Relative	−18.0%	−0.3%	+2.7%	0%	−4.3%
	Absolute	−45.8 Mt	−1.1 Mt	+6.6 Mt	0 Mt	−40.4 Mt
Difference	Absolute	−20.6 Mt	−3.1 Mt	−0.9 Mt	0 Mt	−24.7 Mt
	Share	84%	13%	4%	0%	100%

Some of the period being projected is in fact historical, and if temperature data were available, these could be used to constrain particularly the natural gas projection, which is most clearly correlated to temperature. However, since the natural gas projection has the lowest error, such temperature-correction would add little to the overall projection skill.

### 4.1 Natural gas

The projected growth in the EU's CO₂ emissions from natural gas was +3.0%, while the estimate now reported by the Global Carbon Project is 2.7% (Friedlingstein et al., 2020).

**Fig. 8:**
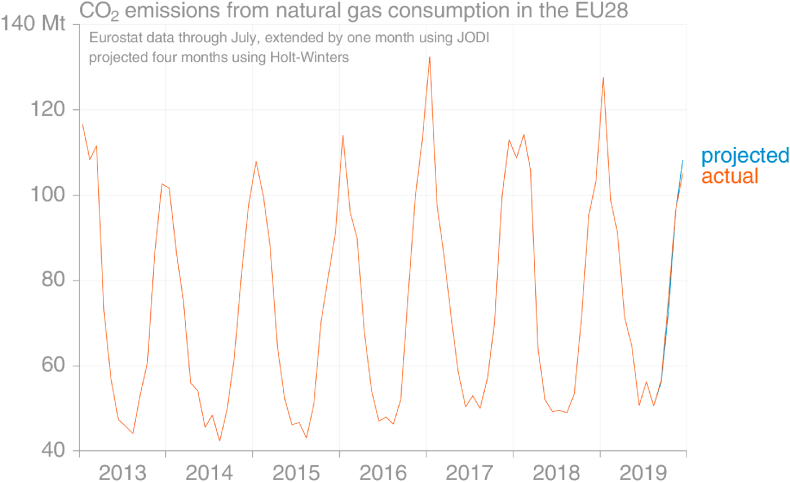
Comparison of the Holt-Winters projection for monthly emissions from natural gas using data that were available in early November 2019 with the data later reported (actual) (source: own calculations based on Eurostat, 2021a).

**Fig. 9:**
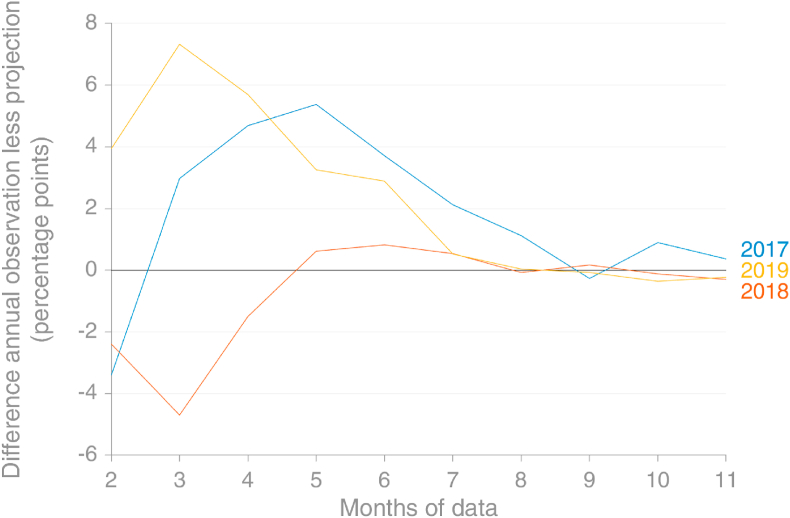
Projection error for the Holt-Winters method versus the number of months of observed data available, EU28, for natural gas. With 8 months of data, HW performs very well in 2017–19 (source: own calculations based on Eurostat, 2021a).

Emissions from consumption of natural gas in the EU show a very strong seasonal signal, with January peaks whose magnitude most likely varies according to temperature more than other factors (Fig. 8). This signal is much more variable for individual countries (not shown), but the size of the EU appears to mitigate some of this variation. Fig. 9 demonstrates that the Holt-Winters method used to extrapolate observed monthly emissions from natural gas would perform relatively well when at least 8 months’ observations in the current year are available, which was how much was available at the time of the projection is made.

Given that the method uses no information about the future apart from an assumption of continued seasonal cycle, it performs very well, a result of natural gas consumption in the EU currently being dominated by a very strong seasonal cycle.

### 4.2 Oil

The projection for growth in 2019 in the EU's CO_2_ emissions from oil was +0.5%, while the most recent estimate reported by the GCP is −0.3%.

**Fig. 10:**
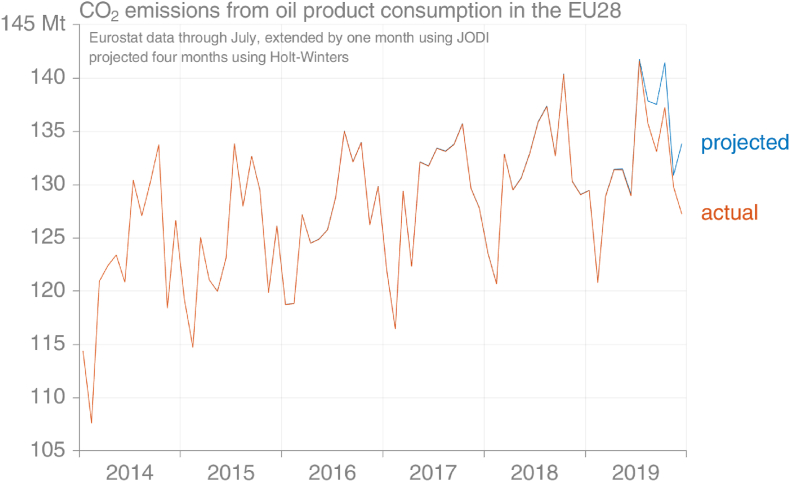
Comparison of the Holt-Winters projection for monthly emissions from oil products using data that were available in early November 2019 with the data later reported (actual) (source: own calculations based on Eurostat, 2021a).

**Fig. 11:**
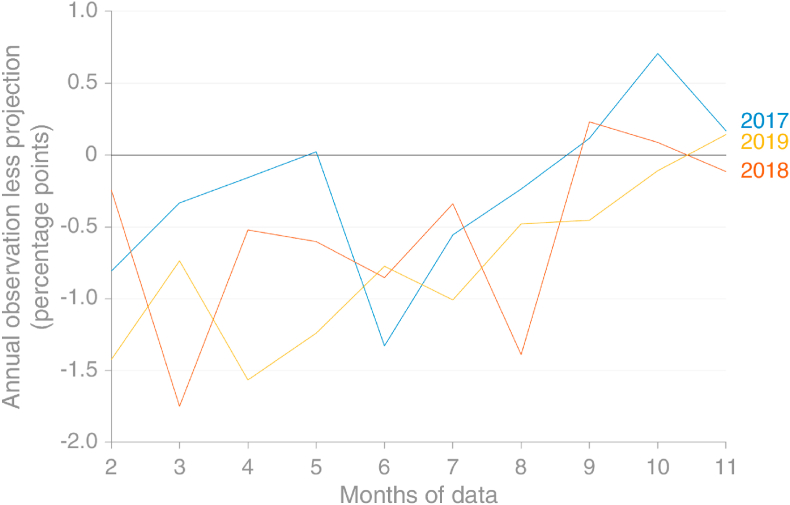
Projection error for the Holt-Winters method versus the number of months of observed data available, EU28 for oil products. With 8 months of data, HW performs very well in 2017–19 (source: own calculations based on Eurostat, 2021a).

It's clear from Fig. 11 that the projection method is less successful for oil products than for natural gas. This is to be expected given the weaker seasonality of emissions from oil products than from natural gas. For example, the Holt-Winters method has ‘learned’ from the data in 2014–2016 that December's emissions from oil consumption are considerably higher than those in November, but the disappearance of this pattern in 2017–2018 is insufficient to fully override this lesson (Fig. 10).

**Fig. 12:**
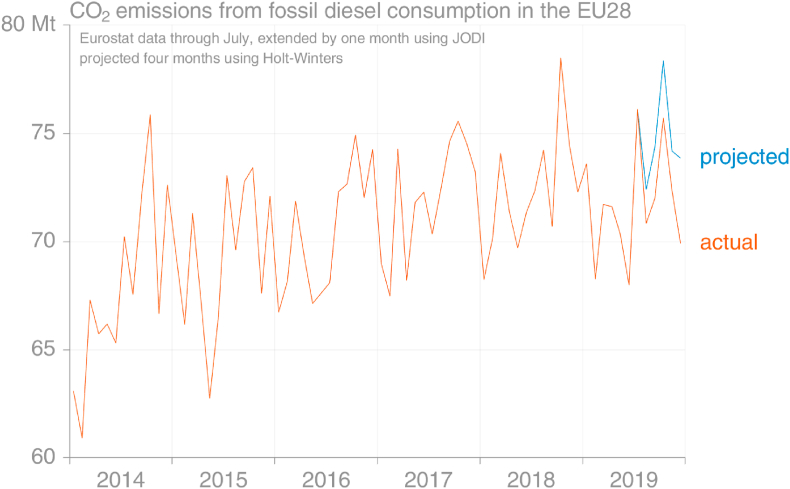
Comparison of the Holt-Winters projection for monthly emissions from fossil diesel using data that were available in early November 2019 with the data later reported (actual) (source: own calculations based on Eurostat, 2021a).

The largest single fuel type in EU consumption is diesel, varying between 52% and 58% of total oil product emissions in recent years. Fig. 12 demonstrates that the seasonal cycle of fossil diesel emissions is relatively unstable, hindering successful projection using seasonality approaches.

### 4.3 Coal

As a result of what was thought to be conflicting information, the mid-point of two approaches was used, giving −10% with large uncertainty, while the estimate now reported by the Global Carbon Project is −18.0% (Friedlingstein et al., 2020) and an estimate using monthly Eurostat data for the full year 2019 also gives −18.0%. The similarity of these two should not cause surprise, since GCP uses a growth rate in the final year taken from BP's energy statistics, which in turn are most likely derived from Eurostat data (BP does not provide sources). Method 1, using monthly Eurostat energy data, gave a projected growth rate of −6%, while Method 2, using electricity generation and steel production data, gave −15%.

Projection method 1 used the monthly coal energy data from Eurostat that were available on November 4, 2019. At that time, most countries had reported data only through July, while Poland – the second-largest coal consumer in the EU at the time – had reported only through May. The projection was based on what was believed to be a parsimonious – ‘least-heroic’ – assumption that the remainder of the year would be the same as the previous year. Fig. 12 clearly shows that this assumption was ill-founded, with estimates made on the data available now showing much lower coal consumption in the latter half of 2019 than in 2018.

**Fig. 13:**
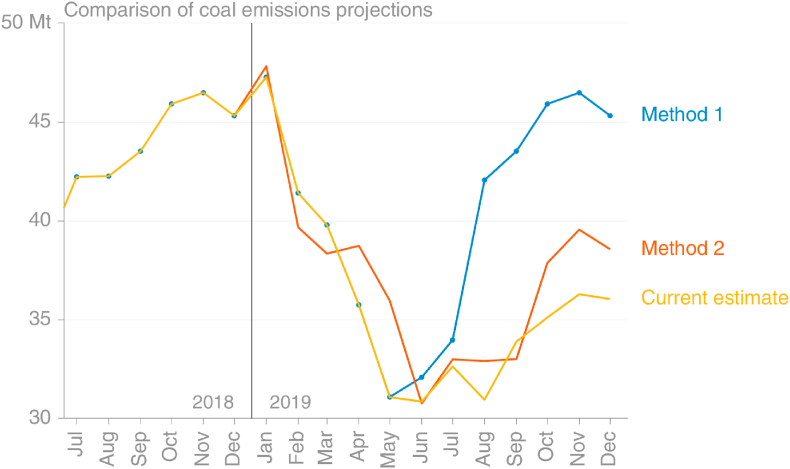
Comparison of projection methods for monthly EU coal emissions. Method 1 using Eurostat data and assuming remaining months identical to 2018. Method 2 using ENTSOE/Steel and assuming remaining months same growth rate as growth rate to date. (source: own calculations based on ENTSO-E, 2021; World Steel, 2020).

The assumption that coal emissions in the remainder of 2019 would be the same as in the same period in 2018 was equivalent to saying that the trend seen in the first months of 2019 was anomalous, and that things would return to ‘normal’ (Fig. 13). This assumption therefore implicitly disregarded information that was available on the downward trend over the previous years. Further, this method disregarded additional information available on coal consumption from the lower-lag datasets on electricity consumption and steel production, which clearly showed reduced consumption in those two important sectors. However, the comparison shown in Fig. 5 of the proxy method using those sectors' weighted data with the method based on Eurostat data had not been performed, and it was unclear how accurate or useful the proxy method would be.

**Fig. 14:**
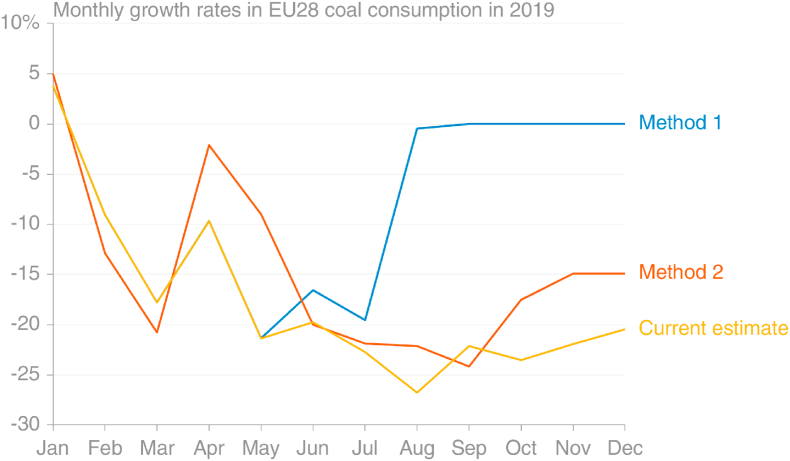
Quantification of the assumptions made in projecting 2019's emissions from coal consumption. Method 1 using monthly data on coal consumption, Method 2 using electricity generation and steel production data (source: own calculations based on Eurostat, 2021a).

Projection method 2 used the lower-lag data on electricity generation and steel production, available through October and September 2019, respectively, at the time the analysis was performed. The projection was based on the assumption that the full year would grow at the same rate as the period covered by observations. This essentially assumed that the final months would grow at the average of the growth rates from January to October, and thereby ignored any seasonal differences in the inter-annual trend, and any decline caused by market conditions. This meant the growth rate in January had as much weight as the growth rate in October in the projection for the full year, but it's clear now that in that space of time the situation had changed markedly (Fig. 13; Fig. 14).

While Method 2, with its use of incomplete proxy data, was imperfect, the much lower lag on the data sources and more sensible projection assumption led to a substantial improvement over Method 1. While in retrospect Method 2 would have been improved by projecting using a shorter period over which to average the growth rate (rather than from January), giving a lower projection for the final 2–3 months, it is difficult to say that this would have been the safest course without the benefit of hindsight.

Also of note is that changing weather conditions, particularly temperature, often play an important role in the analysis of sub-annual energy consumption, but the changes in coal consumption caused by other factors dwarf any weather-related effects.

### 4.4 Cement

Emissions from cement clinker production were projected to remain unchanged in 2019. Data from Eurostat show that cement clinker production declined in 2019 by 0.03%, so the assumption used in the projection turned out to be very good (Eurostat, 2020a).

## 5 Conclusions

The Global Carbon Project (GCP) has been producing short-term projections of the current year's global CO_2_ emissions since 2012, with a sub-projection for the European Union introduced in 2018. The aim is to produce an operational system for relatively reliable projections with minimal intervention. But in 2019 the projection performed poorly, and sufficient official data are now available to perform an assessment and analysis of that projection.

Any short-term projection is reliant first on estimates of recent emissions and second on the method used for projecting those. In this work, therefore, these two stages have been analysed separately. Both stages break total emissions down by fuel type – coal, oil, gas – as well as cement process emissions, although this last category is relatively small in the EU.

The method used to estimate recent emissions was based on monthly energy data collated by Eurostat from reports by member countries. For coal, however, these data had an unsatisfactory lag in several important countries, so a parallel approach was developed using data on electricity generation and steel production. In this work several important errors have been discovered in the sub-annual emissions estimates for both oil and coal, while the method used for monthly emissions from natural gas worked very well.

For oil, three errors were found to be significant. First, the flow used as a proxy for consumption included sales to international aviation bunkers, which should be excluded from national emissions estimates. Second, combustion of primary fuels such as crude oil and natural gas liquids was excluded. Third, naphtha was assumed to be entirely unoxidized, while the actual rate is about 20%. Correcting these three errors resulted in annualised emissions estimates substantially closer to those reported by the GCP. A further source of error is the lack of differentiation of biofuels in JODI data, the effect of which requires further investigation.

For coal, the main error was in the interpretation of the difference in results of the two approaches as being disagreement, when it was in fact largely because they covered different periods. Analysis here has shown that the proxy method – using electricity generated from solid fossil fuels and production of steel – results in a reasonably good approximation of emissions calculated using monthly coal data, but with a much lower lag. This suggests a combination of the two approaches would be optimal.

While errors were found in the methods, no method would be perfect based on monthly energy data because of various reporting problems in Eurostat's data. Further effort will be invested in finding data errors and filling data gaps to mitigate some of these problems.

Different methods were used for projecting the emissions from the three fuel categories. For natural gas, a simple Holt-Winters approach – which extends recent seasonal patterns and trends – performed very well, a result of the relatively steady consumption pattern of natural gas in the EU, which continued without disruption even through the COVID-19 pandemic.

The projection method used for oil also relied on Holt-Winters extrapolation, but with much more variable data. This variability has declined with the corrections made to the observations.

For coal, the projections differed both because of the two apparently conflicting estimates of recent, sub-annual emissions, and because of different assumptions about how trends would continue through the end of the year. The assumptions applied to both methods are shown here to have made poor use of information on recent trends that was available from the observations. In total, 84% of the error in the GCP's 2019 EU CO_2_ projection was because of coal, but this error should be substantially reduced with the corrections made to the sub-annual estimates and future improvements to the assumptions made in projections.

While the goal of an operational, low-maintenance projection method sounds reasonable, the reality is that understanding the reasons behind recent changes in trends and seasonal patterns is important to making short-term projections. Without this understanding, simple assumptions such as “things will continue as they were” or “things will return to normal” are blind to valuable information that aren't contained in the numbers of the time series. Further work is therefore required in operationalising not just the mechanics of estimating sub-annual emissions and projections from these, but also the interpretations of recent emissions trajectories. Many things about the future cannot be known, but information on the recent past should be used as an important guide.

The energy landscape of the EU is undergoing substantial change, particularly affecting the consumption of coal, which has recently become more expensive than other energy sources for electricity generation. The growing presence of renewable generation capacity affects natural gas consumption to a much lesser extent than it does coal consumption. Trajectories for natural gas and also oil products are likely to be more gradual than for coal, at least in the coming 5–10 years, as natural gas is protected by low prices and a buffering effect of substantial coal capacity that is affected first, and oil consumption in transport changes slowly with turnover of the fossil-fuelled vehicle fleet.

The estimates produced using the (revised) methods described herein are reasonable enough to be useful and informative, and the understandings gained from this analysis will be used in future projections by the GCP. While projections are useful as an expectation of what will happen, they are always difficult, and failures are to be expected because many significant deviations – such as the COVID-19 pandemic – cannot be predicted.

## Credit author statement

RMA conceived of the research, collated and prepared the data, performed the analysis, prepared all visualizations, and wrote the article.

## Declaration of competing interest

The authors declare that they have no known competing financial interests or personal relationships that could have appeared to influence the work reported in this paper.
