# Supplementary material for: Towards near real-time, monthly fossil CO2 emissions estimates for the European Union with current-year projections
Source: Atmos Pollut Res. 2021 Dec;12(12):None. doi: 10.1016/j.apr.2021.101229 (PMC8650615; doi:10.1016/j.apr.2021.101229)
Supplement: Multimedia component 1 [file mmc1.docx]

# Supplementary Material

Towards near real-time, monthly fossil CO_2_ emissions estimates for the European Union with current-year projections

Robbie M. Andrew

Table 1: IPCC Default CO_2_ emission factors by fuel (reproduced from Gómez et al., 2006).

| Fuel type | EF (kg CO2 / TJ NCV) |  | Fuel type | EF (kg CO2 / TJ NCV) |
| --- | --- | --- | --- | --- |
| Crude Oil | 73300 |  | Oil Shale and Tar Sands | 107000 |
| Orimulsion | 77000 |  | Brown Coal Briquettes | 97500 |
| Natural Gas Liquids | 64200 |  | Patent Fuel | 97500 |
| Motor Gasoline | 69300 |  | Coke Oven Coke and Lignite Coke | 107000 |
| Aviation Gasoline | 70000 |  | Gas Coke | 107000 |
| Jet Gasoline | 70000 |  | Coal Tar | 80700 |
| Jet Kerosene | 71500 |  | Gas Works Gas | 44400 |
| Other Kerosene | 71900 |  | Coke Oven Gas | 44400 |
| Shale Oil | 73300 |  | Blast Furnace Gas | 260000 |
| Gas/Diesel Oil | 74100 |  | Oxygen Steel Furnace Gas | 182000 |
| Residual Fuel Oil | 77400 |  | Natural Gas | 56100 |
| Liquefied Petroleum Gases | 63100 |  | Municipal Wastes (non-biomass fraction) | 91700 |
| Ethane | 61600 |  | Industrial Wastes | 143000 |
| Naphtha | 73300 |  | Waste Oils | 73300 |
| Bitumen | 80700 |  | Peat | 106000 |
| Lubricants | 73300 |  | Wood / Wood Waste | 112000 |
| Petroleum Coke | 97500 |  | Sulphite Lyes (Black Liquor) | 95300 |
| Refinery Feedstocks | 73300 |  | Other Primary Solid Biomass | 100000 |
| Refinery Gas | 57600 |  | Charcoal | 112000 |
| Paraffin Waxes | 73300 |  | Biogasoline | 70800 |
| White Spirit and SBP | 73300 |  | Biodiesels | 70800 |
| Other Petroleum Products | 73300 |  | Other Liquid Biofuels | 79600 |
| Anthracite | 98300 |  | Landfill Gas | 54600 |
| Coking Coal | 94600 |  | Sludge Gas | 54600 |
| Other Bituminous Coal | 94600 |  | Other Biogas | 54600 |
| Sub-Bituminous Coal | 96100 |  | Municipal Wastes (biomass fraction) | 100000 |
| Lignite | 101000 |  |  |  |

## References

Gómez, D. R., Watterson, J. D., Americano, B. B., Ha, C., Marland, G., Matsika, E., Namayanga, L. N., Osman-Elasha, B., Saka, J. D. K., Treanton, K., and Quadrelli, R.: Stationary Combustion, in: 2006 IPCC Guidelines for National Greenhouse Gas Inventories, edited by: Eggleston, S., Buendia, L., Miwa, K., Ngara, T., and Tanabe, K., IGES, Japan, 2006. <http://www.ipcc-nggip.iges.or.jp/public/2006gl/index.html> (Last access: 21 May 2017).
